# Supplementary figures and images for: Strength-dependent perturbation of whole-brain model working in different regimes reveals the role of fluctuations in brain dynamics
Source: PLoS Comput Biol. 2022 Nov 2;18(11):e1010662. doi: 10.1371/journal.pcbi.1010662 (PMC9629648; doi:10.1371/journal.pcbi.1010662)

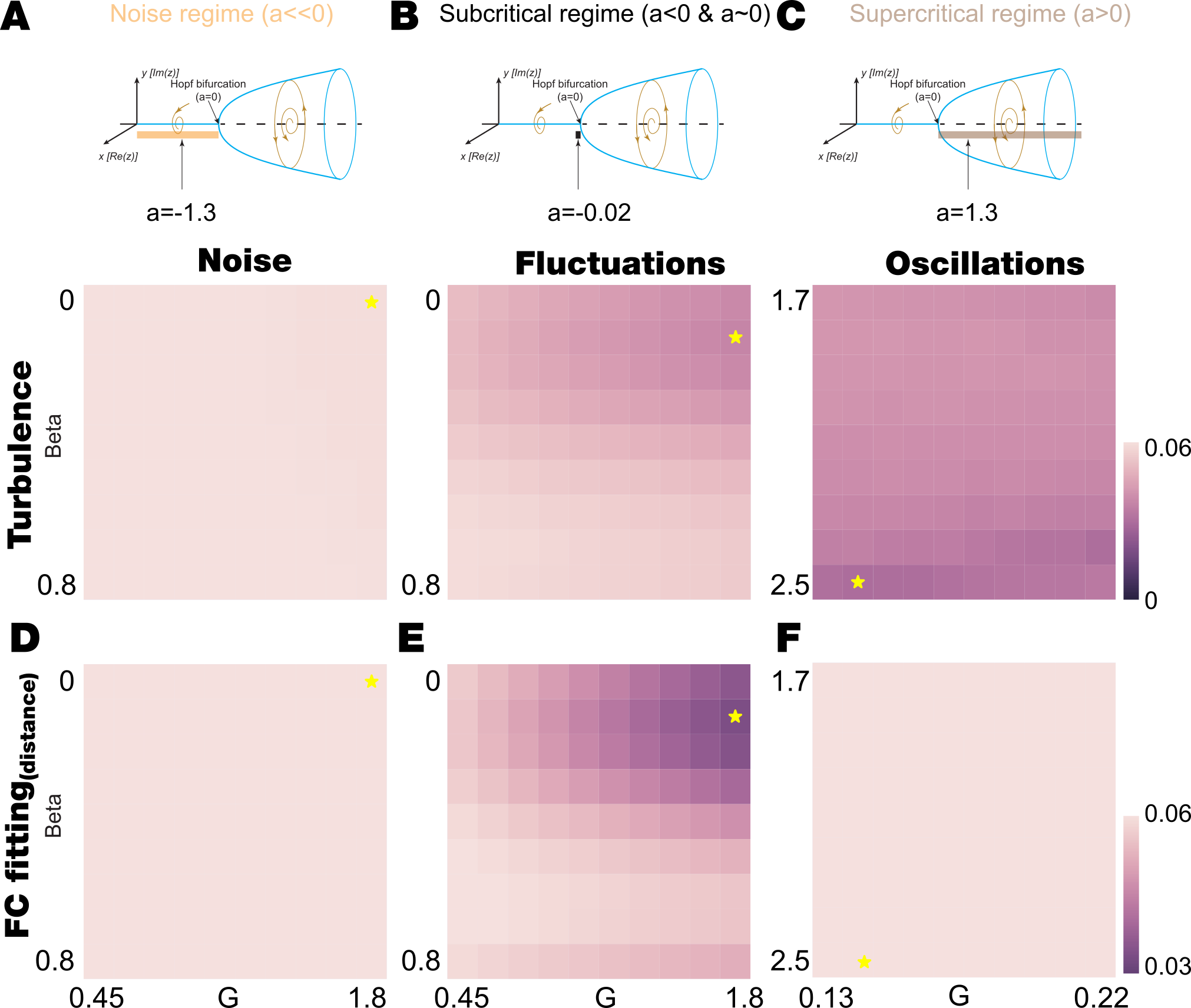

Supplement: S1 Fig — A-C) We explored the bi-dimensional parameter space defined by β and G for noise, fluctuating and oscillatory regime as we performed in Fig 2 (bifurcation parameter a = -1.3, a = -0.02 and a = 1.3, respectively, indicated in upper row). We computed the level of amplitude turbulence error as the absolute difference between the empirical and simulated turbulence. Yellow stars indicate the (β, G) combination that reaches the lowest turbulence error in each regime. E-G) We explored the bi-dimensional parameter space defined by β and G for noise, fluctuating and oscillatory regime computed the FC fitting as Euclidean distance between the empirical and simulated FC. Yellow stars indicate the (β, G) combination that reaches the lower turbulence error in each regime (the optimal working point obtained in panels A-C). (TIF) [file pcbi.1010662.s001.tif]

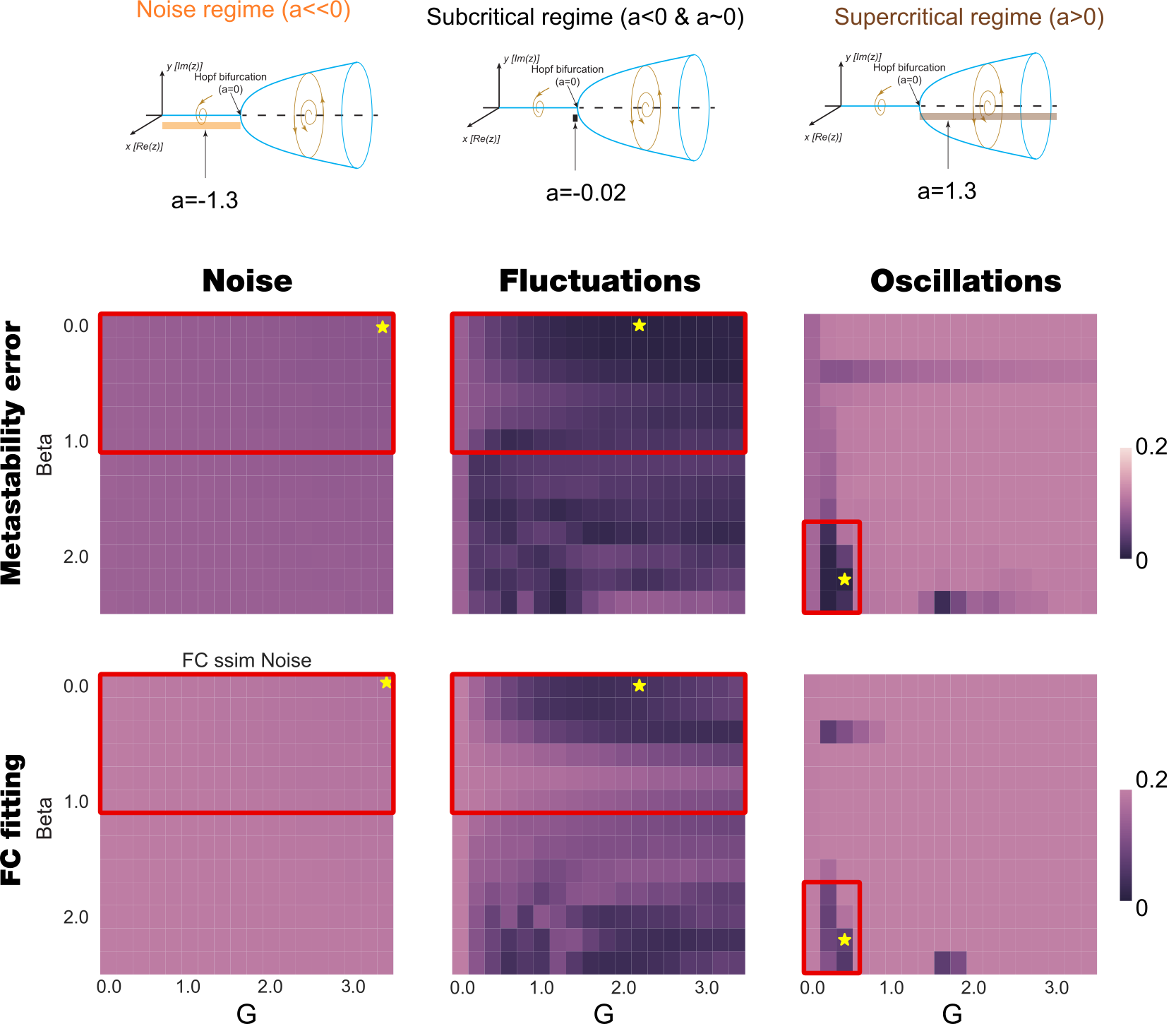

Supplement: S2 Fig — We explored an extended the bi-dimensional parameter space defined by β and G equally for noise, fluctuating and oscillatory regime (bifurcation parameter a = -1.3, a = -0.02 and a = 1.3, respectively, indicated in the upper row) and computed the level of metastability error as the absolute difference between the empirical and simulated metastability. Yellow stars indicate the (β,G) combination that reaches the lowest metastability error in each regime (second row). We explored the bi-dimensional parameter space defined by β and G for noise, fluctuating and oscillatory regime computed the FC fitting as Euclidean distance between the empirical and simulated FC (third row). Yellow stars indicate the (β,G) combination that reaches the lowest metastability error in each regime (the optimal working point obtained in panels second row). We explored the same grid for the three regimes for comparison indicating with red squares the region that were computed in Fig 3. (noise regime were not computed in Fig 3 but we did include in this figure). We confirm that the regions explored in Fig 3 are the optimal working point zones in fluctuating and oscillation regimes. (TIF) [file pcbi.1010662.s002.tif]

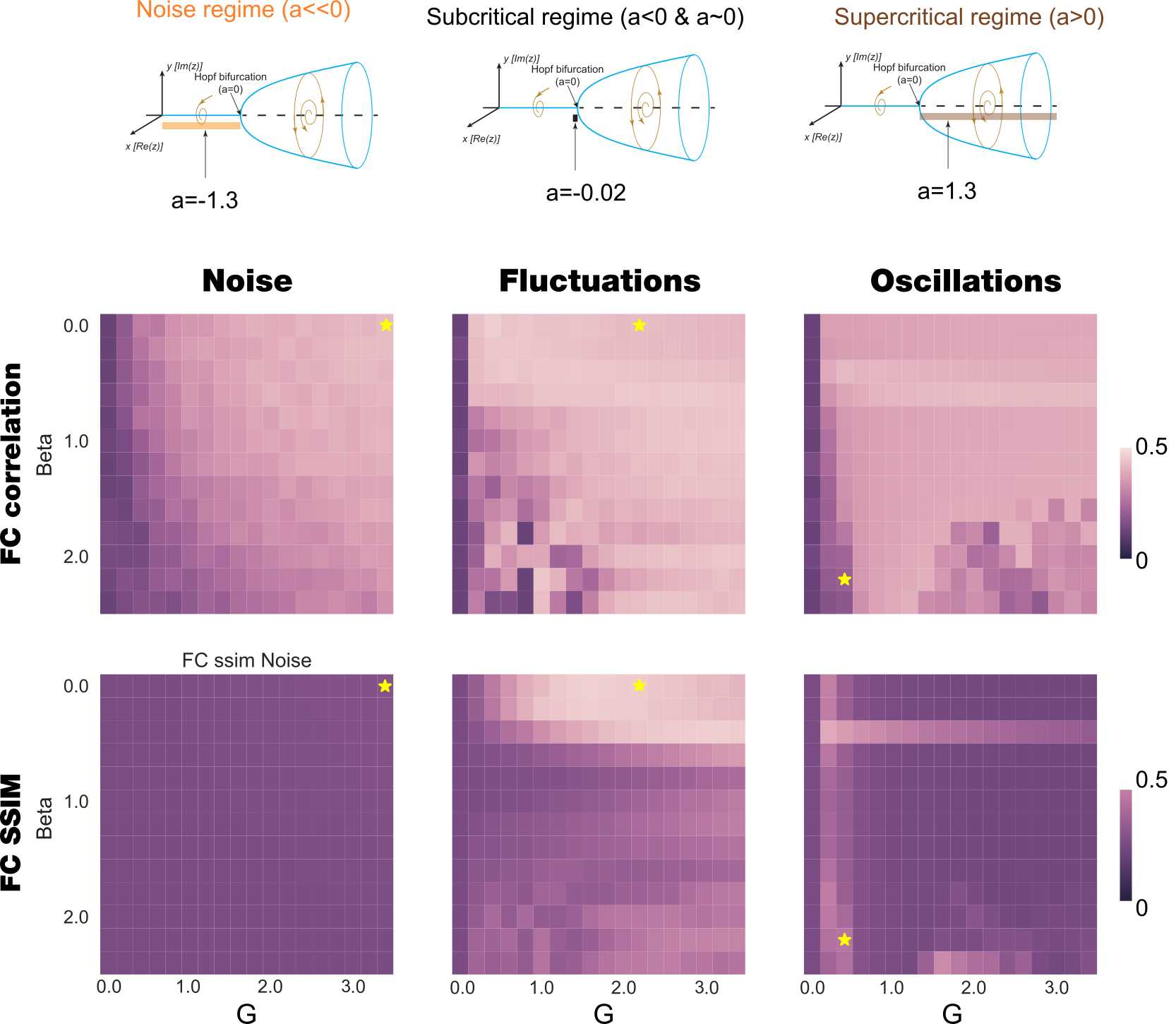

Supplement: S3 Fig — We explored the extended bi-dimensional parameter space defined by β and G for noise, fluctuating and oscillatory regime (bifurcation parameter a = -1.3, a = -0.02 and a = 1.3, respectively, indicated in the upper row) and computed the Pearson correlation (second row) and SSIM (third row) between the empirical and simulated FC. Yellow stars indicate the (β,G) combination that reaches the lowest metastability error in each regime from S2 Fig. (TIF) [file pcbi.1010662.s003.tif]

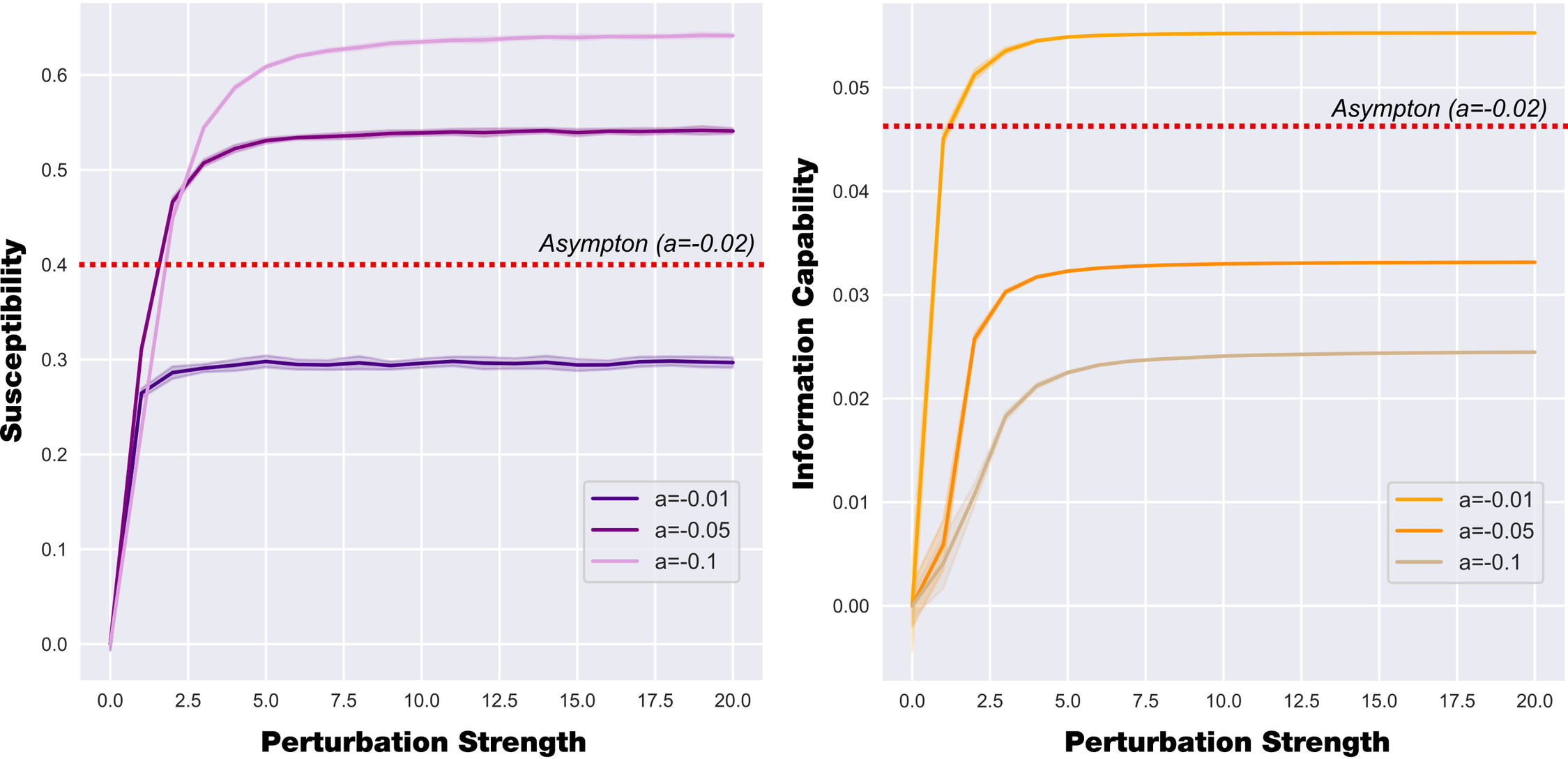

Supplement: S4 Fig — We estimated the Susceptibility and Information Capability of the model at it optimal working point when is perturbed as a function of the perturbation strength in Desikan-Killiany parcellation. We found that when the value of a is closer from the bifurcation point (a = 0), the Susceptibility increases (left panel) and the Information Capability decreases (right panel). Red lines stand for the asymptotic values reached for a = -0.02 displayed in Fig 4. (TIF) [file pcbi.1010662.s004.tif]
